# Economic Burdens for Treatment of Patients With Type 2 Diabetes in North Thailand: A Hospital-Based Observational Study

**DOI:** 10.3389/fendo.2022.824545

**Published:** 2022-05-16

**Authors:** Arintaya Phrommintikul, Piyameth Dilokthornsakul, Unchalee Permsuwan

**Affiliations:** ^1^ Department of Internal Medicine, Faculty of Medicine, Chiang Mai University, Chiang Mai, Thailand; ^2^ Center for Medical and Health Technology Assessment (CM-HTA), Department of Pharmaceutical Care, Faculty of Pharmacy, Chiang Mai University, Chiang Mai, Thailand; ^3^ Center of Pharmaceutical Outcome Research, Department of Pharmacy Practice, Faculty of Pharmaceutical Sciences, Naresuan University, Phitsanulok, Thailand

**Keywords:** economic, cost, complication, cardiovascular complication, Thailand, type 2 diabetes

## Abstract

**Purpose:**

Diabetes and its complications pose an economic burden to healthcare systems, family, and society. Therefore, this study aimed to estimate the real-world financial burden of type 2 diabetes (T2D) treatment, complications, and cardiovascular death.

**Materials and Methods:**

An electronic database of the largest university-affiliated hospital in the North of Thailand was retrieved for a 10-year period (2009-2019). We used the International Classification of Disease 10^th^ Revision codes of diabetes and complications to obtain relevant patient records. All included records based on the inclusion and exclusion criteria were analyzed. Expenditures for diabetes treatment, complications, and cardiovascular death for two years were reported as mean, standard deviation, median, and interquartile range.

**Results:**

Of a total of 9,161 patient records, the average age of patients was 57.8 ± 12.7 years. The average total outpatient cost was THB 22,874 ± 38,066 (US$ 759 ± 1,264) for the first year and THB 23,462 ± 34,441 (US$ 779 ± 1,143) for the second year. The average inpatient expenditure was THB 160,790 ± 411,607 (US$ 5,338 ± 13,666) for the first year and THB 181,804 ± 190,257 (US$ 6,036 ± 6,317) for the second year. Drug was the main component for outpatient expenditure while surgery was the main component for inpatient expenditure. Diabetes patients with complications incurred a greater cost of treatment than those without complications. Cardiovascular death led to about seven times higher cost of treatment than the average total cost of diabetes treatment. Heart failure complications (THB 846,345 ± 752,884 or US$ 28,099 ± 24,996) had the highest inpatient costs compared with other complications in the first year. Stroke complications (THB 71,927 ± 143,414 or US$ 2,388 ± 4,761) had the highest outpatient costs compared with other complications. In general, the first-year expenditure was higher than the second year for all complications.

**Conclusions:**

Diabetes incurs a substantial financial burden resulting from its complications. Effective management of diabetes with a multi-sectoral effort from government, providers, patients, and private is required.

## Introduction

Thailand is an upper-middle-income country in Southeast Asia with gross national income per capita growing from $4,580 USD in 2010 to $7,050 USD in 2020. Life expectancy in 2010 was 74 and increased to 77 in 2019 (1). The extension in life expectancy has contributed to a change in the age distribution of the Thai population. With the increasing age of the elderly population, disease patterns and causes of death have shifted toward non-communicable diseases, which are estimated to account for 71% of all deaths (2). Of those deaths, 27% were cardiovascular, 12% were cancers, and 6% were diabetes (3).

Previous Thai National Health Examination Surveys have shown that diabetes prevalence has been increasing dramatically over time. For diabetes patients aged 20 years and over, the prevalence increased from 7.1% in 2004 to 7.5% in 2009, and to 9.7% in 2014. The proportion of diabetes unawareness declined from 66% in 2004 to 33% in 2009, whereas the proportions of treatment and control for all diabetes increased from 15% in 2004 to 31% in 2009 (4). The increased prevalence of type 2 diabetes (T2D) is likely to be related to multiple factors including environmental factors that affect the lifestyle, especially unhealthy dietary patterns and decreased physical activity levels (4).

Diabetes can lead to many serious health problems, usually after a number of years and particularly if diabetes is not detected early or well treated. The most common diabetic complication is nephropathy at 43.8%, followed by diabetic retinopathy at 30.7%, ischemic heart disease at 8.1% and cerebrovascular disease at 4.4% (5). In addition, T2D presented in about 40% of patients with atherosclerotic cardiovascular disease (6). As the number of people with diabetes and its complications arises, the disease takes a large proportion of national healthcare expenditure. Complications are the main cost driver for diabetes because they require more intensive care such as hospitalization and multiple surgeries. Patients with diabetic complications incurred higher cost of treatment compared to those without complications. However, the magnitude of the difference is varied site by site. Pongcharoensuk P, et al. reported that diabetic complications in patients with diabetes would lead to almost six times higher average cost of treatment compared to those without complications ($1,611 USD vs $281 USD) (7). Another study reported the median cost of diabetic patients with complications in comparison to those without complications was $480 USD vs $115 USD, respectively (8). Preventing complications and related disability by improving diabetes control is therefore of paramount importance to reduce the health and economic burden of diabetes. Although advances in diabetic treatment have improved cardiovascular and renal outcomes (9–11), the access to new treatments is limited in low- and middle-income countries (6, 12). For new diabetic treatment to be listed in the national list of essential medicine, robust cost data and proven cost-effectiveness need to be evaluated for local application.Hence, this study aimed to estimate the real-world economic burden of T2D treatment and its complications in local Thai context.

## Methods

This study is a retrospective database analysis to estimate healthcare resources and financial burden incurred in patients with T2D. Electronic database from Maharaj Nakorn Chiang Mai Hospital, which is the largest university-affiliated tertiary care hospital in the North of Thailand, was retrieved for 10 years.We used the International Classification of Disease 10^th^ Revision (ICD-10) as a primary diagnosis coding E10-E14 to retrieve patient records from the years of 2009 to 2019. The index date was the first visit to a hospital (either in an inpatient or outpatient setting) as aforementioned ICD-10.

Patient records that met the following criteria would be included into the analysis. Firstly, patients were ≥18 years of age and diagnosed with T2D. Secondly, patients should have one year of recorded data without a visit of related T2D in that period. We used a one-year period as a washout period to obtain the incident diabetes cases. Next, patients must have the data of at least a two-year follow-up period after the index date. This was the objective of this study to estimate financial consequences for two consecutive years. Finally, patients must have another visit after the index date within the next six months. This was to confirm consistent visits to this hospital. Other patient records that did not meet the above inclusion criteria were excluded from data analysis.

Once the T2D patient records had been selected, we retrieved the database of diabetes-related complications and cardiovascular death that occurred after the index date using the following ICD-10 codes. Both primary and secondary diagnoses of patient visit with the following diabetes-related complications were included into the analysis. Those complications and ICD-10 codes were myocardial infarction (I21, I22), angina (I20), congestive heart failure (I50), stroke (I64), peripheral vascular disease (I73), neuropathy (G56-64, (E10-E14).4), diabetic retinopathy (E10.3, E11.3, E12.3, E13.3, E14.3, H360), transient ischemic attack (G45, G45.8, G45.9), renal failure (N17, N19), chronic renal failure (N18), hypoglycemia (E16.0-E16.2)), lactic acidosis (E87.2), ketoacidosis ((E10-E14).1), gangrene (R02), and ulcer (L97). In addition, amputation complications were also included using the International Classification of Disease 9th Revision-Clinical Modification (ICD-9-CM) as 8400-8419.

### Data Analysis

We analyzed the healthcare resources in terms of the number of outpatient visits and the number of hospital admissions. Total cost of diabetes treatment, diabetes-related complications, and diabetes-related cardiovascular death was analyzed and reported as mean (standard deviation) and median (interquartile range, IQR). Costs of diabetes treatment were disaggregated into individual cost items such as drugs, laboratory, services, operations, food, and room and reported for two consecutive years. In addition, attributable costs related to T2D with and without complications were estimated for two consecutive years. We started counting the date of complications when thoe T2D patient record reported either primary or secondary diagnosis of ICD-10 of aforementioned complications. Then, costs of outpatients and inpatients were analyzed dividing into those T2D with or without complications. For admitted T2D patients with cardiovascular complications which had a discharge status as dead, all inpatient costs incurred at such admission were analyzed and defined as a cost of cardiovascular death. All costs were inflated by the consumer price index with the medical care section (13), and presented in the year of 2019. Costs in this study were the expenditure or economic burden incurred to the patients or charge. All costs were converted to US$ at the rate of 1 US$ = 30.12 THB (14).

## Results

**Figure 1:**
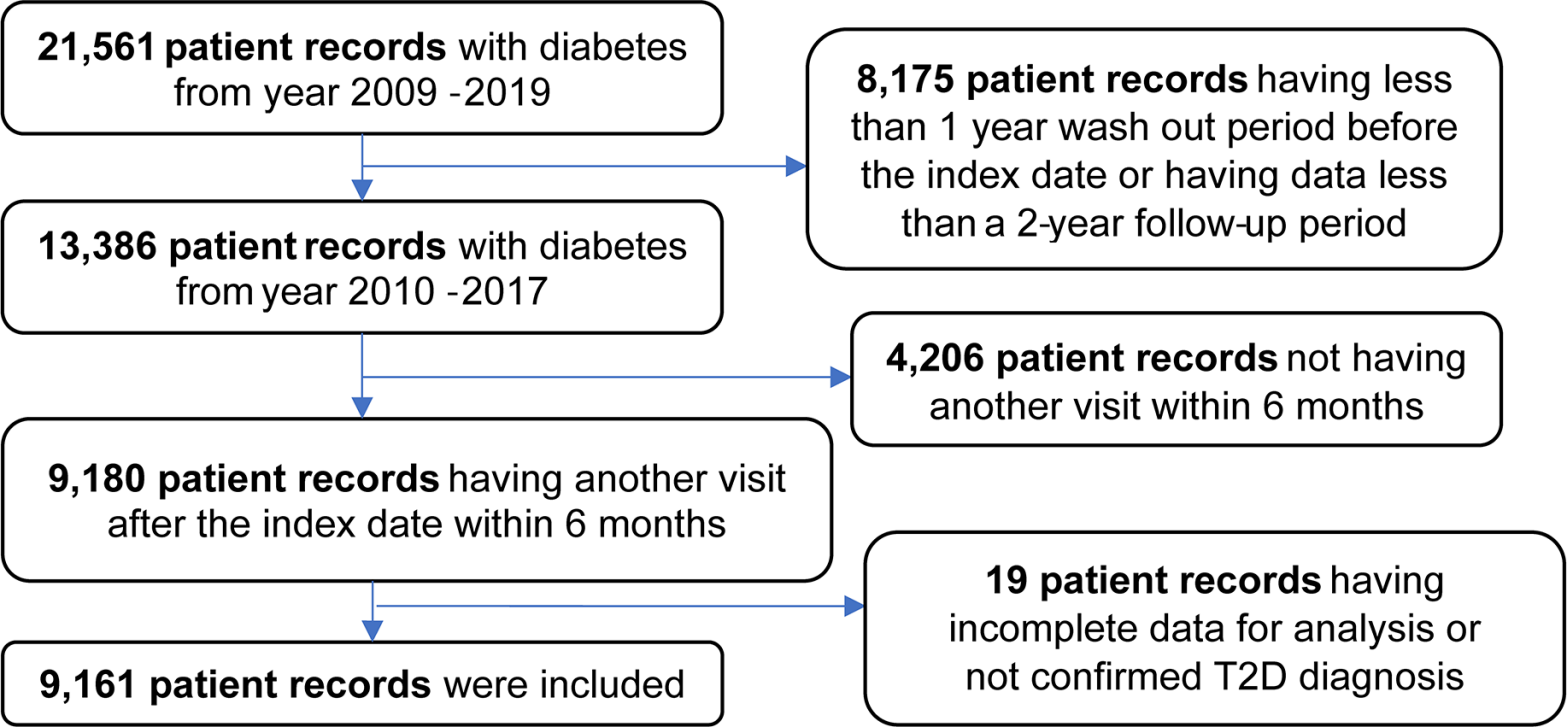
Selection of patient records based on inclusion criteria.

**Figure 1** shows the flow diagram of included patient records for analysis. A total of 21,561 patient records from 2009 to 2019 were retrieved from the electronic database of the Maharaj Nakorn Chiang Mai Hospital. Of the total patient records, 8,175 records were excluded due to having less than one year wash-out period from the index date or having less than a two-year follow-up period. Next, 4,206 patient records were excluded due to not having a second visit within six months after the index date. The remaining 9,180 patient records were initially included into the data analyses. However, 19 patient records were incomplete; therefore, they were excluded from the data analyses. The final of 9,161 T2D patient records were used for data analyses.

From a total of 9,161 patient records, patient’s mean age was 57.8 ± 12.7 years and 53.2% were female. The majority of them (49.6%) had the health insurance of the Civil Servant Medical Benefit Scheme (CSMBS) (**Table 1**).

**Table 1:** Baseline characteristics.

Demographics	N (%)
Number of included patient records	9,161
Age (mean, standard deviation)	57.8 ± 12.7
Gender	
Male	4,290 (46.8)
Female	4,871 (53.2)
Health insurance scheme	
Civil servant medical benefit scheme	4,540 (49.6)
Universal coverage scheme	2,467 (26.9)
Social security scheme	1,637 (17.9)
Others	517 (5.6)

Of all included patient records, 9,161 T2D patients visited the outpatient department in the first year and 5,330 T2D patients revisited in the second year. Costs of the outpatient visits for T2D patients were shown as mean (standard deviation, SD) and median (interquartile range, IQR). The average number of outpatient visit was 5.87 (3.93) for the first year and 5.19 (3.98) for the second year. The mean (SD) of total outpatient costs was THB 22,874 (38,066) or US$ 759 (1,264) and THB 23,462 (34,441) or US$ 779 (1,143) for the first and second year, respectively. The median (IQR) of total outpatient costs was THB 10,210 (3,699;26,872) or US$ 339 (123;892) for the first year and THB 10,827 (3,935;29,731) or US$ 359 (131;987) for the second year. Of the overall total cost, drug cost was the major component, followed by operation and laboratory costs.

Of all included patient records, 445 unique T2D patients were admitted in the first year and 136 patients were admitted in the second year. However, the 136 unique patients in the second year could be the same or different patients as those admitted in the first year. The average number of admissions was 2.70 (3.14) and 3.63 (3.02) per patient during the first and second year, respectively. The mean (SD) total cost per admission was THB 160,790 (411,607) or US$ 5,338 (13,666) for the first year and THB 181,804 (190,257) or US$ 6,036 (6,317) for the second year. The median (IQR) of total cost per admission was THB 85,818 (54,527;151,452) or US$ 2,849 (1,810;5,028) for the first year and THB 112,486 (65,071;225,377) or US$ 3,735 (2,160;7,483) for the second year. Surgery was the main cost contribution, followed by drug and operation. The detail of outpatient and inpatient costs is shown in **Table 2**.

**Table 2:** Economic burden of type 2 diabetes treatment.

Variables	First year of treatment (THB/US$)		Second year of treatment (THB/US$)	
	Mean (SD)	Median (IQR)	Mean (SD)	Median (IQR)
**Outpatient treatment (all included patients (N = 9,161 for first year and N = 5,330 for second year)**				
Number of visits per year	5.87 (3.93)	5 (3-7)	5.19 (3.98)	4 (3-7)
Total cost (THB)	22,874 (38,066)	10,210 (3,699-26,872)	23,462 (34,441)	10,827 (3,935-29,731)
(US$)	759 (1,264)	339 (123-892)	779 (1,143)	359 (131-987)
Drug (THB)	17,843 (37,259)	3,552 (174-19,872)	19,967 (33,229)	6,845 (1,443-25,502)
(US$)	592 (1,237)	118 (6-660)	663 (1,103)	227 (48-847)
Laboratory (THB)	1,605 (2,413)	654 (0-2,514)	1,306 (2,073)	504 (0-1,963)
(US$)	53 (80)	22 (0-83)	43(69)	17 (0-65)
Service (THB)	548 (961)	272 (161-492)	495 (709)	267 (160-508)
(US$)	18 (32)	9 (5-16)	16 (24)	9 (5-17)
X-ray (THB)	214 (1,700)	0 (0-0)	115 (1,296)	0 (0-0)
(US$)	7 (56)		4 (43)	
Operation (THB)	2,235 (7,215)	0 (0-377)	1,237 (6,516)	0 (0-144)
(US$)	74 (240)	0 (0-13)	41 (216)	0 (0-5)
Food (THB)	19 (572)	0 (0-0)	27 (836)	0 (0-0)
(US$)	0.63 (19)		1 (28)	
Other (THB)	409 (1,415)	0 (0-0)	315 (1,410)	0 (0-0)
(US$)	14 (47)		10 (47)	
**Inpatient treatment (only admitted patients; N = 445 for first year and N = 136 for second year)**				
Number of admissions per year	2.70 (3.14)	2 (1-3)	3.63 (3.02)	3 (2-5)
Total cost (THB)	160,790 (411,607)	85,818 (54,527-151,452)	181,804 (190,257)	112,486 (65,071-225,377)
(US$)	5,338 (13,666)	2,849 (1,810-5,028)	6,036 (6,317)	3,735 (2,160-7,483)
Drug (THB)	26,129 (149,179)	5,236 (2,307-11,476)	35,495 (79,047)	6,194 (2,614-28,941)
(US$)	868 (4,953)	174 (77-381)	1,178 (2,624)	206 (87-961)
Laboratory (THB)	9,750 (63,635)	206 (0-1,717)	12,657 (25,434)	739 (51-13,145)
(US$)	324 (2,113)	7 (0-57)	420 (844)	25 (2-436)
Service THB)	16,656 (68,406)	5,650 (2,825-12,364)	18,213 (25,938)	8,827 (5,340-21,617)
(US$)	553 (2,271)	188 (94-410)	605 (861)	293 (177-718)
X-ray (THB)	3,062 (11,090)	0 (0-283)	5,115 (12,150)	274 (0-2,546)
(US$)	102 (368)	0 (0-9.40)	170 (403)	9 (0-85)
Operation (THB)	23,389 (79,165)	9,361 (5,289-18,231)	24,759 (33,927)	11,893 (6,099-25,801)
(US$)	777 (2,628)	310.79 (175.60-605.28)	822 (1,126)	395 (202-857)
Food (THB)	107 (887)	0 (0-0)	123 (758)	0 (0-0)
(US$)	4 (29)		4 (25)	
Room (THB)	9,928 (36,910)	2,476 (1,273-5,657)	12,776 (30,681)	3,981 (1,650-12,368)
(US$)	330 (1,225)	82.20 (42.26-187.82)	424 (1,019)	132 (22-411)
Surgery (THB)	68,149 (73,970)	54,669 (33,483-86,321)	68,005 (50,940)	60,271 (35,374-91,130)
(US$)	2,263 (2,456)	1,815.04 (1,111.65-2,865.90)	2,258 (1,691)	2,001 (1,174-3,026)
Other (THB)	3,619 (17,011)	772 (377-1,780)	4,660 (13,290)	1,130 (553-2,701)
(US$)	120 (565)	25.63 (12.52-59.10)	155 (441)	38 (18-90)

IQR, interquartile range; SD, standard deviation.

Of all 880 patient visits with cardiovascular cause (ICD code: Ixx.xx), 27 admitted patients were discharged with dead status. Therefore, inpatient costs incurred from this patient group were estimated as the cost of cardiovascular death. We found that the mean cost (SD) was THB 193,596 (270,929) or US$ 6,427 (8,995). The median with IQR was THB 90,108 (35,836;227,515) or US$ 2,992 (1,190;7,554).

**Figure 2:**
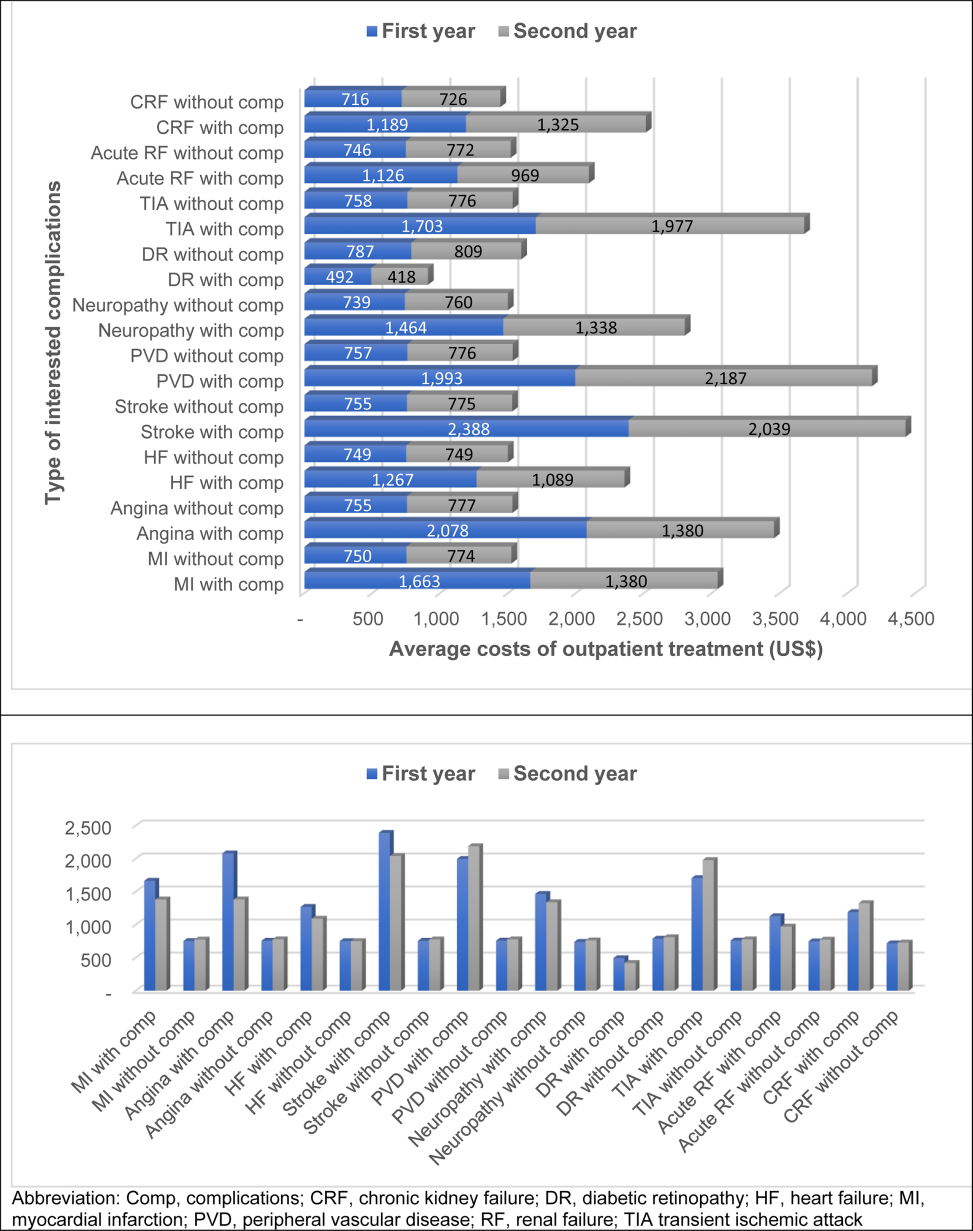
Outpatient treatment costs for type 2 diabetes with and without interested complications.

Of all 9,161 T2D patients who visited the outpatient department in the first year, diabetic retinopathy was the highest number of complications found, followed by renal failure, neuropathy, and heart failure (850, 312, 263, 181 patients, respectively). T2D patients with complications usually incurred greater cost of outpatient treatment than those without complications, except for diabetic retinopathy (**Figure 2**). Among T2D patients with complications, those with stroke incurred the highest average cost of treatment (THB 71,927 or US$ 2,388), followed by angina (THB 62,596 or US$ 2,078), and peripheral vascular disease (THB 60,042 or US$ 1,993). The average outpatient treatment cost of T2D without complication was about THB 22,000-23,000 (US$ 730-764) in the first year. The attributable treatment cost with complication was highest in stroke (THB 49,188 or US$ 1,633), followed by angina (THB 39,870 or US$ 1,324), and peripheral vascular disease (THB 37,233 or US$ 1,236) compared with those without such complications.

Of all 5,330 T2D patients who visited the outpatient department in the second year, the top four complications were similar to those found in the first year. Costs of treatment for T2D patients without complications in the second year were slightly higher than those in the first year. Costs of complication treatment in the second year were lower than those in the first year. There might be several explanations. First, we selected incident cases of T2D, thus, first event of the complications is likely to be more severe than the following events. Therefore, cost of the first year of complication is higher than that of the second year. Second, the number of patients with complications in the first year is higher than that of the second year. Some patients were lost which could be the patient with high treatment cost.The attributable treatment cost with complication was highest in peripheral vascular disease (THB 42,496 or US$ 1,411), stroke (THB 38,078 or US$ 1,264), and transient ischemic attack (THB 36,172 or US$ 1,201). Again, T2D without diabetic retinopathy had greater treatment cost than those with diabetic retinopathy (**Figure 2**).

**Figure 3:**
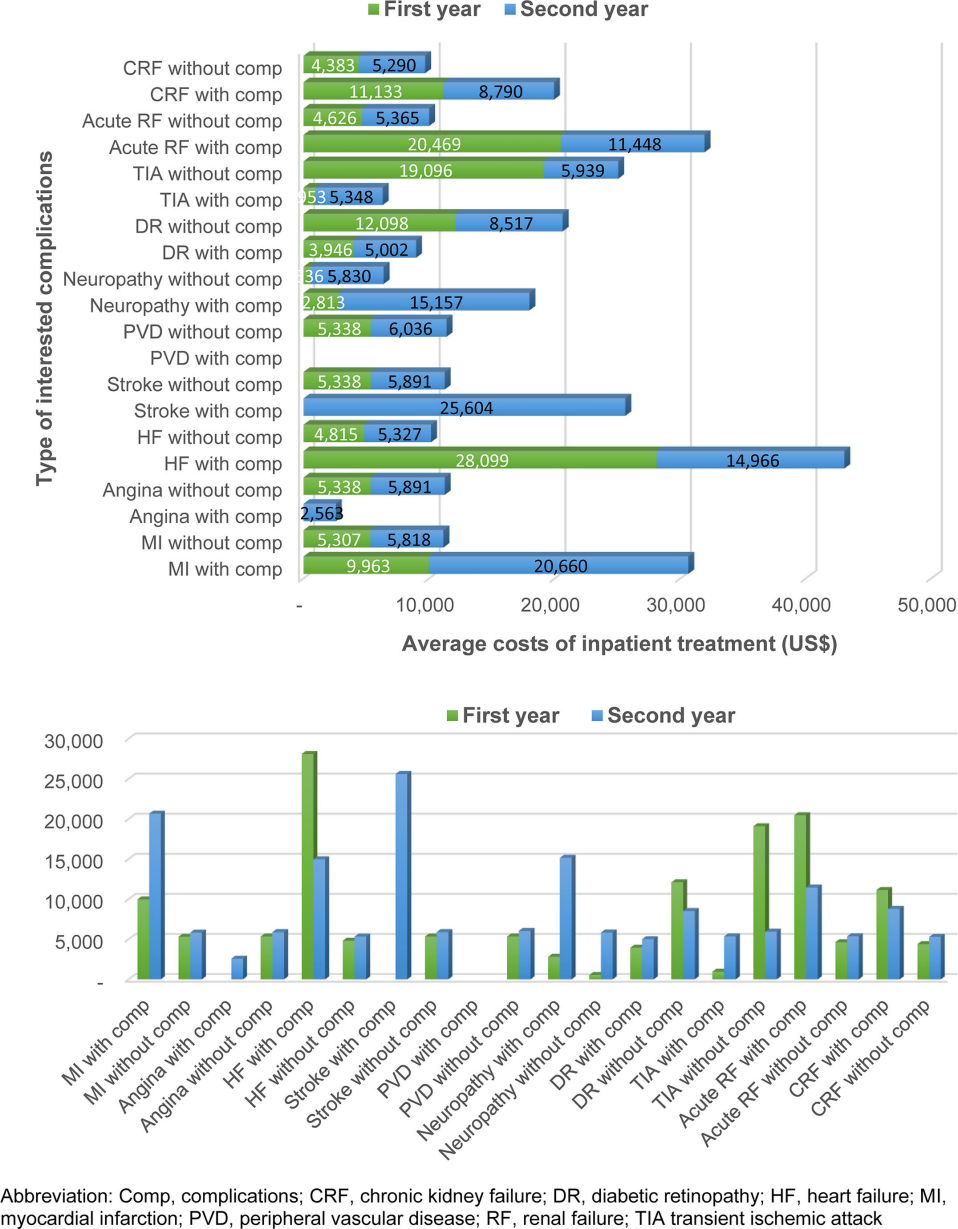
Inpatient treatment costs for type 2 diabetes with and without interested complications.

Of all 445 admitted T2D patients in the first year, diabetic retinopathy was the highest number of complications found, followed by renal failure, and heart failure (369, 20, 10 patients, respectively). There were no T2D patients admitted with stroke, angina, or peripheral vascular disease during the first year; hence, cost of inpatient treatment was not available. Of all 136 admitted T2D patients in the second year, diabetic retinopathy, renal failure, and heart failure were still the top three admission from T2D patients (**Figure 3**).

In general, patients with complications had higher cost of inpatient treatment than those without complications. The difference in treatment cost was substantial, especially in T2D with heart failure compared to those without heart failure (THB 846,345 vs THB 145,031 or US$ 28,099 vs US$ 4,815). T2D patients admitted with heart failure and renal failure incurred the highest cost of treatment (THB 846,345 (US$ 28,099) and THB 616,532 (US$ 20,469), respectively). In the second year, T2D with stroke, angina, or myocardial infarction had greater cost burdens than those without such complications (**Figure 3**).

**Figure 4:**
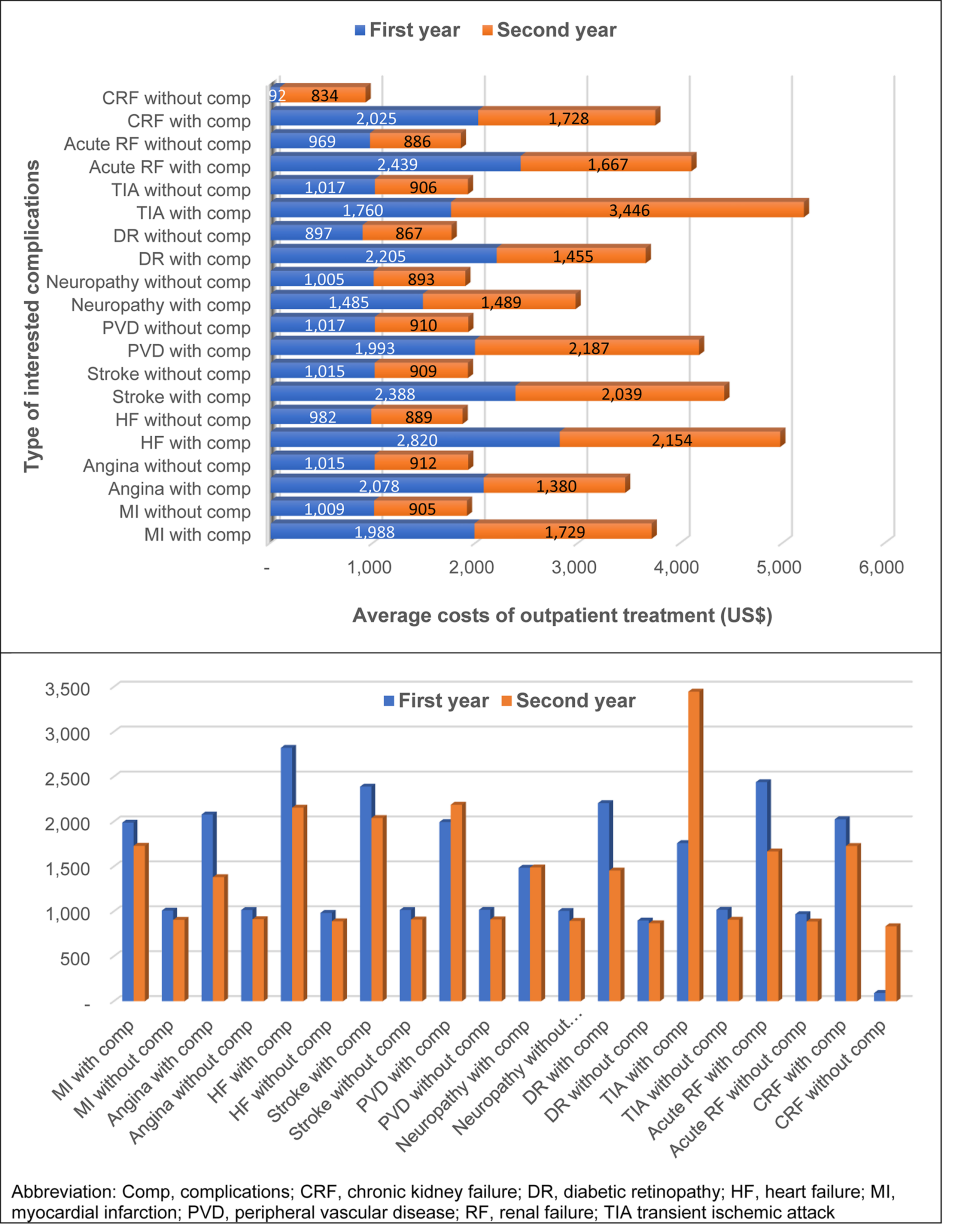
Total treatment costs for type 2 diabetes with and without interested complications.

**Figure 4** shows the total cost of treatment for T2D patients with or without complications. For the first year, T2D patients with complications had incurred higher treatment costs than those without complications. The difference in average total treatment costs ranged from THB 14,472 to 55,344 (US$480 to 1,837). In general, the mean total cost of treatment for T2D patients without complication were about 30,000 THB (US$ 996) per patient per year. Once T2D patients had complications, treatment costs would increase to THB 44,000-84,000 (US$ 1,461-2,789), depending on the type of complication. It was found that heart failure (THB 84,935 or US$ 2,820), renal failure (THB 73,449 or US$ 2,439), and stroke (THB 71,927 or US$ 2,388) were the top three complications that incurred highest treatment costs.

Cost of diabetes treatment in T2D patients without complications was slightly decreased in the second year compared to the first year. Transient ischemic attack (THB 103,787 or US$ 3,446), peripheral vascular disease (THB 65,871 or US$ 2,187), and heart failure (THB 64,875 or US$ 2,154) incurred highest treatment costs for T2D with complications in the second year (**Figure 4**).

**Figure 5:**
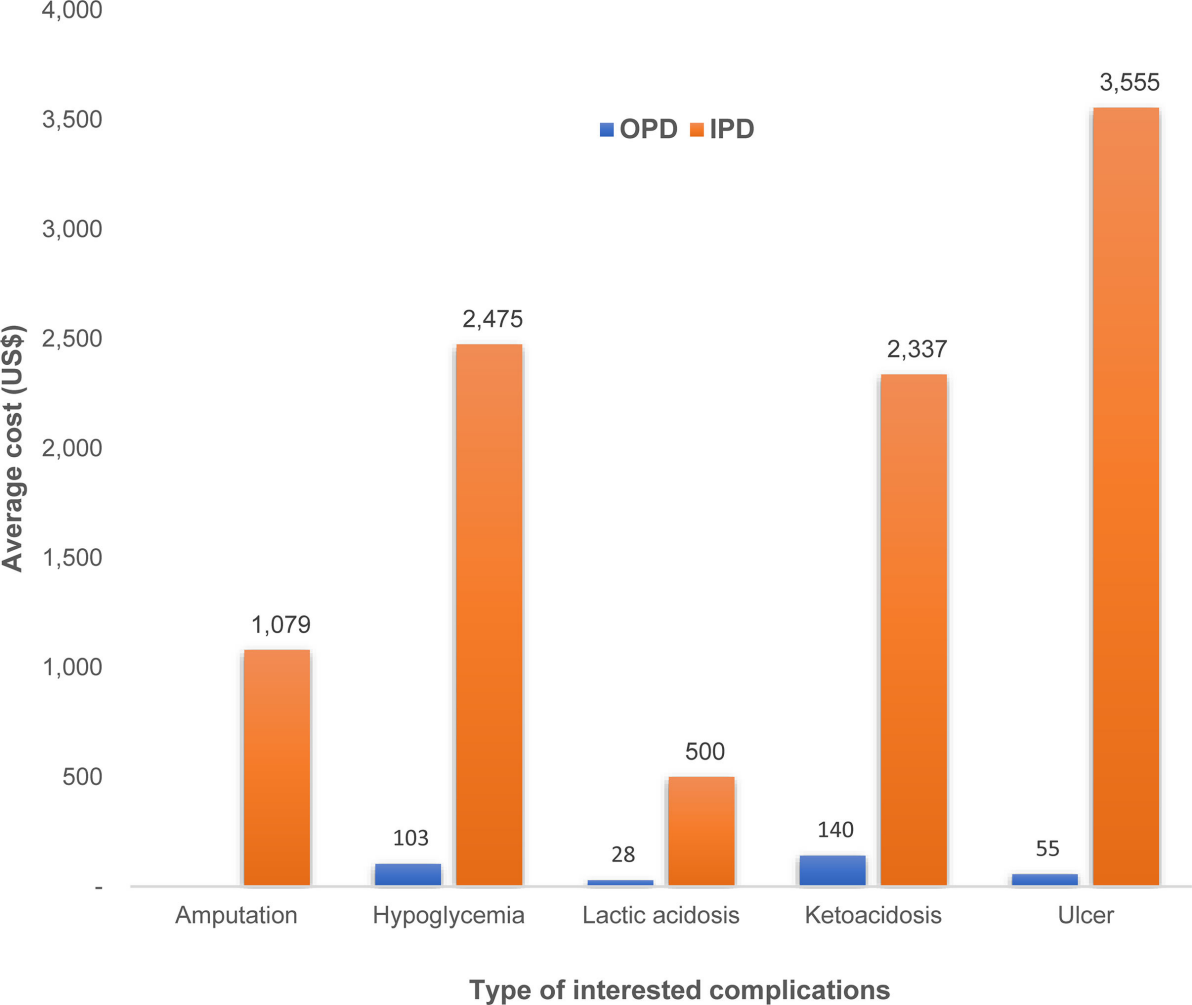
Event-based treatment costs for type 2 diabetes related complications.

We estimated some diabetes complication costs as their event-based costs. Those complications were amputation, hypoglycemia, lactic acidosis, ketoacidosis, and ulcer. Ketoacidosis had the highest number of outpatient visit and highest cost of outpatient treatment (THB 4,230 or US$ 140). Hypoglycemia event incurred the second highest cost of outpatient treatment even though the number of outpatient visits was much lower than those with ketoacidosis. In case of inpatient costs, T2D patients with ulcer complication incurred the highest treatment cost (THB 107,067 or US$ 3,555), followed by hypoglycemia (THB 74,532 or US$ 2,475), and ketoacidosis (THB 70,391 or US$ 2,337) as shown in **Figure 5**.

## Discussion

This study aimed to capture real-world evidence of expenditures incurred from diabetes treatment. We used the electronic database from the largest university-affiliated hospital in the North of Thailand. The medical records from the period of 2009 to 2019 were retrieved using the specified ICD-10 codes and were analyzed in terms of financial burden from treatment of diabetes and its complications. We found that the average outpatient treatment cost was THB 22,874 (38,066) or US$ 759 (1,264) and THB 23,462 (34,441) or US$ 779 (1,143) for the first and second year, respectively, in T2D patients with or without complications. Drug costs were the main contribution and accounted for 78% of all outpatient costs. Financial burden was about seven times higher in inpatient treatment than outpatient treatment.The average inpatient cost was THB 160,790 (411,607) or US$ 5,338 (13,666) in year 1 and THB 181,804 (190,257 or US$ 6,036 (6,317) in year 2. Approximately 40% of inpatient expenditure was from surgery. Diabetes patients with complications incurred greater financial burden than those without complications. Heart failure, renal failure, and stroke were of important complications that led to financial burden for T2D patients. Cardiovascular death from T2D patients had the average cost of THB 193,596 (US$ 6,427).

Cost of diabetes care has been estimated in several studies in Thailand. A small study conducted in 475 diabetic patients found that the median cost for patients without complications was lower than those with complications (US$ 115 vs US$ 479, respectively) (15). A larger study of 24,501 diabetic patients reported US$ 551 for diabetes cost of care (16). The average spending per admission in a tertiary care hospital in Bangkok, the capital, was estimated at US$ 1,682 (17). Therefore, the estimation of diabetes care costs was varied depending on the hospital care levels, presence of complications, among other factors.

The findings of this study were somewhat different from the findings from another study (18) that obtained the economic burden from the database of the largest tertiary hospital, Buddhachinaraj hospital, located in the lower North of Thailand. This might be due to different healthcare contexts in each setting such as physicians and patients. Maharaj Nakorn Chiang Mai hospital is the university tertiary hospital serving both referred patients around the upper northern Thailand and walk-in patients, while Buddhachinaraj hospital is a tertiary regional hospital serving patients around the lower northern Thailand. The complexity of T2D patients visiting those hospitals might be different leading to different costs of T2D treatment.

Our findings were in line with other studies that have indicated the rising costs of diabetes from complications. A study was conducted in Thailand to estimate the cost of diabetes and its complications using a micro-costing approach. The average cost per diabetic patient was US$ 882 in 2008 (1 US$ = 32 THB) (8). Existence of complications increased the cost substantially (3, 8). The median cost of illness of patients with complications was significantly higher than for people without complications (US$ 479.93 vs US$ 115.12 in 2008) and increased with increasing numbers of complications (19). A study predicted the cost of diabetes to increase up to 232% depending on the type of complications (20).

With the study design of retrospective database analyses, several limitations were taken into consideration. Firstly, patients must not visit a hospital with the diagnosis of diabetes for a year to confirm as an incident case. It is possible that patients are not really an incident case due to loss to follow-up over a year or referral from other hospitals. In such instances, some expenditures might be neglected. Secondly, we cannot definitely conclude that the complications were the result of diabetes although complication data were obtained later than the diabetes data. It is possible that such complications had been found and treated before diabetes was diagnosed. Then, those complications were relapsed or reoccurred after diabetes occurrence. Third, since we are looking at patients identified over a 10-year period, there may have been changes in the treatment guidelines,e.g., greater use of DPP4i in the latter years.

Although this study has aforementioned limitations, it creates some value and has become the source of information for cost-effectiveness study and budget impact analysis. This is because the findings of the study reflect the real-world treatment costs for diabetes as opposed to the protocol driven costs based on restrictive patient inclusion/exclusion criteria, which are used very often in randomized controlled trials. In addition, we obtained almost all cost data from patients who met the inclusion criteria except for 19 incomplete patient records. These findings could be an important input for further cost-effectiveness analysis of new health technology for T2D treatment in Thailand. For example, the cost-effectiveness of sodium-glucose cotransporter 2 (SGLT2) inhibitors for T2D patients with high risk of cardiovascular disease or the cost-effectiveness of SGLT2 inhibitors for T2D patients with chronic kidney disease.

## Conclusion

Diabetes and its complications have posed an economic burden to healthcare system. Average annual expenditure for diabetes treatment was about THB 25,000 (US$ 830). Once complicationsoccurr, they lead to substantial financial burden. Cost of cardiovascular death from T2D patients was about seven times higher than the average annual cost of T2D treatment. Cardiovascular complications, such as stroke, angina, and heart failure were the main drivers of substantial cost of complication treatment. Effective management of diabetes with a multi-sectoral effort from government, providers, patients, and private is required.

## Data Availability Statement

The original contributions presented in the study are included in the article/supplementary materials. Further inquiries can be directed to the corresponding author.

## Ethics Statement

This study was reviewed and approved by the Institutional Research Board of the Faculty of Medicine, Chiang Mai University, Study code: MED-2562-06811.

## Author Contributions

Conceptualization: AP, PD, UP. Data retrieval: AP. Data analysis and interpretation: PD, UP, AP. Project administration: UP. Writing-original draft: UP. Writing-review and editing: PD, AP, UP. All authors read and approved the final manuscript.

## Funding

This study was supported by Boehringer Ingelheim Thailand.

## Conflict of Interest

The authors declare that the research was conducted in the absence of any commercial or financial relationships that could be construed as a potential conflict of interest.

## Publisher’s Note

All claims expressed in this article are solely those of the authors and do not necessarily represent those of their affiliated organizations, or those of the publisher, the editors and the reviewers. Any product that may be evaluated in this article, or claim that may be made by its manufacturer, is not guaranteed or endorsed by the publisher.
